# Behavioural biomarkers and mobile mental health: a new paradigm

**DOI:** 10.1186/s40345-018-0119-7

**Published:** 2018-05-06

**Authors:** Diego Hidalgo-Mazzei, Allan H. Young, Eduard Vieta, Francesc Colom

**Affiliations:** 1 Bipolar Disorder Program, Department of Psychiatry and Psychology, Institute of Neuroscience, Hospital Clinic, University of Barcelona, IDIBAPS, CIBERSAM, Villaroel 170, 08036 Barcelona, Catalonia Spain; 2 Centre for Affective Disorders, Institute of Psychiatry, Psychology & Neuroscience, King’s College London, De Crespigny Park, London, SE5 8AF UK; 3 Mental Health Group, IMIM-Hospital del Mar, Dr. Aiguader 88, 08003 Barcelona, Catalonia Spain

**Keywords:** Biomarkers, Digital markers, mHealth, Smartphones, Psychiatry

## Abstract

Over recent decades, the field of psychiatry has allocated a vast amount of resources and efforts to make available more accurate and objective methods to diagnose, assess and monitor treatment outcomes in psychiatric disorders. Despite the optimism and some significant progress in biological markers, it has become increasingly evident that they are failing to meet initial expectations due to their lack of specificity, inconsistent reliability and limited availability. On the other hand, there is an increasingly emerging evidence of mobile technologies’ feasibility to measure mental illness activity. Moreover, taking into account its widespread use, availability and potential to capture behavioural markers, mobile-connected technologies could be strong candidates to fill and complement—at least at some degree—the gaps that biological markers couldn’t. This represents an especially interesting opportunity to reform our current diagnostic system using a bottom-up research methodology based on digital and biological markers data instead of the classical traditional top-down approach. Therefore, the field might benefit of further exploring this promising –and increasingly evidence-based- pathway as well as other auspicious alternatives in order to attain a more holistic and integrative approach in research, which could ultimately impact real-world clinical practice.

## Main text

Over recent decades, the field of psychiatry has allocated a vast amount of resources and efforts to make available-to both clinicians and researchers-more accurate and objective assessments methods to diagnose, monitor and treat mental disorders (Kapur et al. 2012). These initiatives were driven and justified by the increasing and growing-although scattered-evidence of the biological basis of these illnesses as well as the advancements in related genetic, molecular and cellular techniques and findings. Additionally, this promised a clearer answer to the need of more uniform and operational taxonomy in the field to narrow the gap with other medical disciplines thus possibly opening the pathway to a more precise and tailored research and clinical standards. The subjectivity, imprecision, and lack of granularity of the symptoms reported by individuals until now imposed a limitation that could not be overcome with traditional psychometric methods. Quantifying the qualitative expressions of the brain remains our main and only resource during the diagnosis and treatment of mental illnesses. Beyond the already complicated challenge of traditional interviews, assessments scales and psychometric batteries attempting to capture different degrees of emotions, feelings, thoughts, and behaviours, these instruments also have to deal with the inter-rater, cultural and linguistic differences mining their uniform standardization (Nordgaard et al. 2013). Taking into account these factors, the increasing enthusiasm and hope regarding the short-term availability and full deployment of accurate objective biological assessments in real-world settings and diagnostic manuals were and still are well justified.

Most of this wave of research was focused on identifying biological biomarkers that could accurately detect mental disorders, measure severity thus allowing better staging systems in order to provide more tailored treatments, with response promptly evaluated. Despite the optimism and some significant progress in genetic and neuroimaging approaches, in recent years, it has become increasingly evident that the pathway of biological biomarkers alone was failing to meet initial expectations due to their lack of specificity, inconsistent reliability and limited availability (Venkatasubramanian and Keshavan 2016). Not surprisingly, they were left out of recent diagnostic classifications and do not yet reach real-world clinical or research settings. Recently, systematic reviews and meta-analyses evaluated the likelihood of biases in the current scientific literature about peripheral biomarkers of major psychiatric disorders (i.e. depressive, bipolar and psychotic disorders). Most of them concluded that there is a general overestimation of statistical significance among the publications evaluated due to selective positive results reporting and publication biases (Carvalho et al. 2016a, b; Prata et al. 2014).

Beyond the potential bias and limitations, the accumulated evidence regarding peripheral biomarkers still has a crucial role in uncovering the pathological underpinnings of psychiatric conditions. However, considering this background and the lessons learnt from the biomarkers endeavour, it is persistently evident that a more holistic and comprehensive approach might be needed to get to the bottom of these complex mechanisms (Scarr et al. 2015).

Almost at the same time and in parallel to the aforementioned research efforts, the miniaturization, and interconnectedness of powerful and complex digital devices over the internet, has led to its wide availability and use in almost every daily activity of individuals of all ages, localities and ethnicities around the world. These mobile devices are carried all day long ubiquitously by most of us and are fully capable of capturing detailed unbiased insights into users’ behaviour and thoughts. The massive data continuously collected by increasingly consumer-affordable smartphones and wearables, are now frequently used by companies for marketing and commercial purposes as well as for tracking individuals’ well-being. On the top of that, smartphones are progressively capturing most of the individual social activity—a key factor in most mental disorders—as digital social networks and messaging services complement or even replace a significant percentage of personal face-to-face contacts (Abdullah et al. 2016; Pierce 2009).

In view of this scenario, it is timely to think of the potential implications of these technologies for mental health, now denominated mHealth. Many projects have already tested the feasibility and reliability of these “digital behavioural biomarkers” in major mental health disorders with encouraging results using phone usage patterns and device sensors (Faurholt-Jepsen et al. 2016a, b). Many others have used smartphones or discrete wearables devices-or both-capable of capturing in detail physical activity, sleep patterns and circadian rhythms successfully—also critical and intrinsic parameters in most mental disorders—which until now were only roughly assessed subjectively by most clinicians and researchers. Most of these studies have demonstrated significant levels of correlations with mental illnesses symptoms (De Crescenzo et al. 2017; Scott et al. 2017). Hence, these new and widely available technologies provide an authentic and continuous source of individuals’ behavioural markers or digital footprints (Insel 2017). This represents a paradigm change in the field and a realistic bridge narrowing the gap between behaviour and psychopathological phenomenology underlying mental illnesses instead of the more pretentious gap distance from biological mechanisms.

Moreover, although evidence is growing that internet-connected devices are increasingly owned, used and adopted by patients in the same way of the general population (Conell et al. 2016), there is a general sense of disbelief and scepticism about the potential of these technologies in the scientific community. Notwithstanding, the number of publications and studies so far evaluating these technologies severely lacks behind peripheral biomarkers in major psychiatric disorders. For instance, a quick search on PubMed and Clinicaltrials.gov shows that among all the publications and registered trials exploring mental disorders objective markers, only 1–5% respectively, pertain or include digital markers as for 2017. Very few study protocols so far have incorporated these technologies alongside biological parameters to study the course of major psychiatric illnesses. Currently, only a few European research projects in the field [i.e. R-Link, RADMIS (Faurholt-Jepsen et al. 2017) and BIO cohort (Kessing et al. 2017)] are going to employ these complementary mHealth methods alongside other biological markers in the longitudinal follow-up of affective disorders patients.

Unquestionably a rigorous process of ethical, safety, quality, and effectiveness standardization still lie ahead before these technologies can be fully deployed in clinical and research grounds whilst minimizing the potential for patients to be exploited by the digital economy. A key concern is that information from patients unaware of the terms and conditions could potentially be sold, combined and utilized by second- or third-party companies to rate, classify or categorize individuals. Subsequently, results might be misused by commercial and government institutions alike to assess eligibility for health, economic and social privileges resulting in indirect harm to patients (Bauer et al. 2017). Thus, comprehensive, realistic and flexible guidelines, are urgently needed to help standardize, ensure patient’s safety and reach a consensus on how to develop, evaluate and validate these new, but diverse methods, in both academic and commercial projects (Hidalgo-Mazzei et al. 2015b).

Yet, as the evidence is gradually suggesting, these mobile technologies could be strong candidates to fill and complement-at least at some degree—the gaps that biological markers couldn’t in the complex and intricate etiopathogenic mechanisms underlying mental disorders while simultaneously serving as multifaceted tools for treatment interventions (Hidalgo-Mazzei et al. 2015a). This represents an especially interesting opportunity to reform our diagnostic system using a bottom-up research methodology based on digital and biological markers data instead of the current top-down approach centred on our limited classification systems. Promising progress in Big data analytics and machine learning could further disentangle this still difficult maze to extract meaningful mental disorders’ onset, course and treatment predictors (Monteith et al. 2016; Torous et al. 2016). Even if it might not be the source of all the answers, the field should consider distributing its efforts and resources to explore this promising –and increasingly evidence-based- pathway as well as other auspicious alternatives towards a more holistic and integrative approach instead of concentrating all prospects in a limited number of research lines.
